# Evaluating the temporal and situational consistency of personality traits in adult dairy cattle

**DOI:** 10.1371/journal.pone.0204619

**Published:** 2018-10-01

**Authors:** Borbala Foris, Manuela Zebunke, Jan Langbein, Nina Melzer

**Affiliations:** 1 Institute of Genetics and Biometry, Leibniz Institute for Farm Animal Biology, Dummerstorf, Mecklenburg-Western Pomerania, Germany; 2 Institute of Behavioural Physiology, Leibniz Institute for Farm Animal Biology, Dummerstorf, Mecklenburg-Western Pomerania, Germany; University of Illinois, UNITED STATES

## Abstract

Recent research suggests that personality, defined as consistent individual behavioral variation, in farm animals could be an important factor when considering their health, welfare, and productivity. However, behavioral tests are often performed individually and they might not reflect the behavioral differences manifested in every-day social environments. Furthermore, the contextual and longer-term temporal stability of personality traits have rarely been investigated in adult dairy cattle. In this study, we tested three groups of lactating Holstein cows (40 cows) using an individual arena test and a novel object test in groups to measure the contextual stability of behavior. Among the recorded individual test parameters, we used seven in the final analysis, which were determined by a systematic parameter reduction procedure. We found positive correlations between novel object contact duration in the group test and individual test parameters object contact duration (R_s_ = 0.361, *P* = 0.026) and movement duration (R_s_ = 0.336, *P* = 0.039). Both tests were repeated 6 months later to investigate their temporal stability whereby four individual test parameters were repeatable. There was no consistency in the group test results for 25 cows tested twice, possibly due to group composition changes. Furthermore, based on the seven individual test parameters, two personality traits (activity/exploration and boldness) were identified by principal component analysis. We found a positive association between the first and second tests for activity/exploration (R_s_ = 0.334, *P* = 0.058) and for boldness (R_s_ = 0.491, *P* = 0.004). Our results support the multidimensional nature of personality in adult dairy cattle and they indicate a link between behavior in individual and within-group situations. The lack of stability according to the group test results implies that group companions might have a stronger influence on individual behavior than expected. We suggest repeating the within-group behavioral measurements to study the relationship between the social environment and the manifestation of personality traits in every-day situations.

## Introduction

Recently, there has been a growth in interest in the connections between personality, health, welfare, and productivity in farm animals [1,2]. It has been suggested that different personalities may vary in terms of their disease susceptibility [3], physiological response to stress [4,5] and production traits [6–11]. Furthermore, considering personality in the context of animal breeding seems to be a promising approach for improving the robustness and welfare of farm animals [1,12]. Personality is applied as a term in many species to refer to individual behavioral variation that is stable across time and context [13,14]. However, the term temperament is often used when referring to farm animals, probably to avoid anthropomorphism [15–17]. In the present study, we use the term personality trait to refer to “a particular aspect of an individual’s behavioral repertoire that can be quantified and that shows between-individual variation and within-individual consistency” [18]. The framework proposed by Réale et al. [19] generally considers five personality traits in animals: activity, exploration, boldness, sociability and aggressiveness (similar to the “Big-Five factor model” used in humans [20]). There is still debate regarding whether these personality traits are exclusive and if they can be assessed in all species [21].

Several studies have assessed the multidimensional character of personality in calves (e.g. [22–24]) and multiple personality traits have been reported. However, personality traits and their stability might change throughout ontogenesis [25,26] and the contextual and longer-term temporal stability of personality traits in adult lactating cows has rarely been investigated (but see [27,28]). Personality is commonly assessed using individual tests, including social isolation (runway test), novelty (open-field, novel object), or fear eliciting situations (forced human approach) [29,30]. At present, little is known about whether the individual test parameters measured in previous studies reflect the behavioral differences manifested in the every-day lives of dairy cattle. Gibbons et al. [31] found a connection between the sociability of dairy cows measured in an individual runway test and behavioral measures of sociability in the home pen. Furthermore, MacKay et al. [28] found a relationship between neophobia and boldness in dairy cows measured in a novel arena and novel object test and their lying behavior in the home pen, which were derived from longer term tri-axial accelerometer data. These studies indicate a certain level of behavioral consistency between individual and group contexts.

Ohl and Putman [32] argued that in a social species, the welfare of an individual depends on the welfare of its group companions to some extent. In addition, it has been suggested that the personality of individuals may play a role in the formation and maintenance of animal social networks [33], and thus personality could also be a relevant factor when considering the social welfare of dairy cattle groups [34]. Therefore, given the possible link between individual personality and the welfare of the group, it is important to assess the manifestation of personality traits within a group context. Furthermore, recording robust behavioral parameters in a practical manner (i.e., a simple test in the home pen instead of laborious individual tests) might facilitate routine assessments of personality in future animal husbandry.

Therefore, in this study, we first aimed to investigate whether the behavioral parameters assessed in a traditional individual test using lactating Holstein cows could be captured in a more practical test performed under group housing conditions. To assess the contextual stability of behavior, we applied a novel object test, which was performed as an individual test and also in the home pen group. In addition, the temporal stability of the behavioral parameters was determined in repeated individual and group tests 6 months later. We also used selected individual test parameters to derive personality traits. We repeated this procedure after 6 months to evaluate whether the identified personality traits exhibited temporal stability, which is necessary for potential practical application.

## Animals, materials, and methods

### Animals and housing

Individual and within group behavioral tests were performed on adult lactating Holstein-Friesian cows in spring 2016 (March–April; parity range: 1–3, age range: 2.3–5.2 years, days in milk range: 11–509) and the tests were repeated in autumn 2016 (October–November; parity range: 1–4, age range: 2.3–5.2 years, days in milk range: 4–589). The cows were housed in three separate groups (in spring: G_1_ = 11 cows, G_2_ = 14 cows, G_3_ = 15 cows; in autumn: G_1_ = 12 cows, G_2_ = 14 cows, G_3_ = 11 cows) in a loose housing barn at the Leibniz Institute for Farm Animal Biology (FBN, Dummerstorf, Germany). Each group area (21.5 × 7.5 m) contained 15 deep litter lying stalls with straw, two electronic water bins, and 10 electronic feed bins (Insentec RIC System, Hokofarm Group, Marknesse, Netherlands) where the total mixed ration was provided ad libitum. In all three groups every cow had access to all bins in the group and cows were slightly overstocked at feed bins. In the individual test situation, 39 cows were tested in the spring and 33 cows were retested in the autumn. In the group test situation 40 cows were tested in the spring, 38 of which also participated in the individual test, due to experimental reasons. In the autumn, 25 of the 40 cows were retested in the group test (G_1_ = 7 retested cows, G_2_ = 8 retested cows, G_3_ = 10 retested cows). The group composition changed slightly between spring and autumn mainly because cows left for the dry period and returned after calving. All of the cows were healthy and not in heat during the behavioral tests. All animal care and experimental procedures were performed in accordance with the German welfare requirements for farm animals and the ASAB/ABS Guidelines for the Use of Animals in Research [35]. All procedures involving animal handling and treatment (repeated individual and group behavioral tests) were approved by the Animal Welfare Committee of the Leibniz Institute for Farm Animal Biology (FBN) and by the Committee for Animal Use and Care of the Ministry of Agriculture, Environment and Consumer Protection of the federal state of Mecklenburg-Western Pomerania, Germany (Mecklenburg-Western Pomerania State Office for Agriculture, Food Safety, and Fishery; Reference number: 7221.3-2-033/15).

### Experimental procedure

#### Individual test

We performed a combined arena test in a closed observational arena, which was previously unknown to the cows. In the arena (5 × 10 m) the cows did not have any visual or auditory contact with conspecifics, because it was located in a separate, sound-isolated building close to the barn. The arena contained a one-way mirror on one side to allow the supervision of the experiment from an adjacent room (cows could only see it from 5 m but they could not approach it) and the flooring of the arena was made of rubber mats without bedding. We assigned each cow to one of the nine test days based on a randomized design to facilitate statistical testing for known fixed effects (i.e., age and parity; see S1 Table). The arena test was performed on each test day between 7:00 am and 12:00 pm, as follows. A familiar person led a cow from the barn to the arena. The combined arena test comprised three consecutive parts: 1) a novel arena test (NA) where the cow spent 10 min alone in the arena; followed by 2) a novel object test (NO) (Part A in S1 Fig) where an unknown object was lowered down from the ceiling and this was removed after 10 min; directly followed by 3) a novel human test (NH) where an unknown human in standardized clothing (white overalls, which were unknown to the cows and not used by the barn staff) entered and stood at the predefined positionin the arena for 10 min. The arena was cleaned with a scraper between tests and with high pressure water at the end of the test day. Tests were recorded with two video cameras (Sony YC 3189, Sony Corp., Tokyo, Japan) installed at opposite ends of the arena and with a digital recorder (EDR HD-2H14/4H4, EverFocus Electronics Corp., New Taipei City, Taiwan). During the combined arena test, 73 behavioral parameters were recorded, as suggested in previous studies (see [29] for a review). The recorded behavioral parameters and their definitions are provided in Table 1. We did not record play behavior (commonly used parameter in calves) because it has not been observed during the test. Vocalization was recorded using the audio channel of the video recordings.

**Table 1:** Behavioral parameters recorded during the arena tests.

Recording type	Novel arena test	Novel object test	Novel human test	Definition
*D*, F, L, MD		*Object Look*	*Human Look*	Looking at the object/human
*D*, F, L, MD		*Object Contact*	*Human Contact*	Actively touching the object/human
*D*, F, L, MD	*Movement*	Movement	Movement	Taking steps, walking or jumping
*D*, F, L, MD	No movement	No movement	No movement	Standing still, legs not moving
*D*, F, L, MD	*Exploration*	Exploration	Exploration	Sniffing the wall or the floor of the arena
*D*, F, L, MD	*Mirror*	Mirror	Mirror	Looking in the direction of the one-way mirror
F	Urination	Urination	Urination	Urinating
F	Defecation	Defecation	Defecation	Defecating
F	Vocalization	Vocalization	Vocalization	Vocalizing
No. test parameter	19	27	27	

The recorded parameters and recording types for each part of the arena test are shown. Recording types: duration (D) in s, frequency (F), latency (L) in s, and mean duration (MD) in s. The behavioral parameters and the corresponding types used for further analyses are shown in italics.

#### Group test

We performed a novel object test with each group [36] in their home pen during the spring and autumn, as follows. A novel object was hanging in the middle of the walking alley (21.5 × 3.65 m) for 3 h (8:00–11:00 am) and the area around the object (Part B in S1 Fig) was recorded with a camera (Panasonic HDC-SD 600, Panasonic Corp., Osaka, Japan). The latency (s), duration (s) and frequency of active contacts with the novel object were determined as behavioral parameters for each cow.

#### Settings and video analysis

In the individual and group tests, we changed the form but kept the color and size (~30 cm diameter) of the objects used constant in the test repetitions. We used a yellow round bowl and a rectangular tray in the individual test, and an orange-black ball and can in the group test. The colors used in both test contexts were similar (see S1 Fig) and visible to the cows [37]. Video data were coded using Mangold Interact v15 (Mangold International GmbH, Arnstorf, Germany). All the video coding was conducted by one trained observer.

### Statistical analysis

All of the analyses were performed in R version 3.4.2 [38] unless specified otherwise. The significance level was set to *P* < 0.05.

#### Behavioral parameters

Our goal was to retain the recorded parameters that possibly reflected true individual behavioral differences in the reaction to novelty in our setup, and to reach the 5 animals to parameter ratio which is suggested as a minimum when applying principal compent analysis (PCA) [39]. Hence we excluded behavioral parameters according to the following conditions: 1) high level of possible external influence (by discarding defecation, urination, and vocalization); 2) potential bias caused by the experimental setup (by discarding latencies due to possible discrepancies between test start and start of a behavior, and discarding environment related parameters in the NO and NH tests since in these tests the environment is not novel anymore); 3) high interdependency with other parameters (by discarding all recording types with the behavioral parameter “No movement”); and 4) small between-animal variability (by discarding the recording types with the mean duration and frequency for all parameters). Finally, to ensure that measurement types remained consistent between tests, we used seven behavioral parameters from the individual test (shown in italics in Table 1) and the object contact duration from the group test for further analysis.

In a preliminary analysis we tested whether the retained behavioral parameters are influenced by known effects. The effects of test day, age and parity were considered for the individual test parameters (duration of movement, exploration, mirror, object look, object contact, human look, and human contact) whereas the effects of group, age and parity were analyzed for the group test parameter (duration of object contact). The spring and autumn data sets were analyzed separately in view to the differences between cows based on the following known fixed effects (S1 Table shows the raw data): 1) test day (1–9 in spring and autumn); 2) parity (1–3 in spring and 1–4 in autumn); and 3) age in days (in spring: parity 1: 830–859, parity 2: 1341–1903, parity 3: 1551–1841; in autumn: parity 1: 1034–1048, parity 2: 1703–2037, parity 3: 1586–2155, parity 4: 1793–2064). We observed that the ages of cows were very similar in the first parity, in contrast to the multiparous cows. Hence, only multiparous cows were considered to investigate the impact of age. In this analysis, we applied a linear model where the covariates were age, parity, and test day nested in parity. The fixed effects were tested with an F-Test, where age had no significant effect. Based on these results and due to the high similarity in age of the first parity cows, we excluded the covariate age from the analyses that considered all cows. Thus, the final linear model used to test all the individual test parameters included parity and test day nested in parity. The group test parameter object contact duration (see S2 Table for the raw data) was tested in a similar manner. Age had no impact for multiparous cows, so the final linear model only included the fixed effects of parity and group nested in parity. The final analysis was performed for all cows in spring and for the retested cows in autumn. The linear model analyses were performed in SAS version 9.4 (SAS Institute Inc., Cary, NC, USA) using the PROC MIXED function. We applied the post-hoc Tukey–Kramer test to correct for multiple testing.

**Stability between contexts:** We investigated whether the behavioral parameters measured in the time consuming individual test corresponded to the behavioral parameter object contact measured in the group test, which is a more practical parameter to measure. We hypothesized that there would be positive relationship between object contact duration in the group test and the individual test parameters: exploration, object contact, and human contact. First, the Spearman's rank correlation coefficients were calculated between the individual test parameters and the group test parameter in spring. We considered correlations: R_s_ ≤ 0.40 weak, 0.40 < R_s_ ≤ 0.80 moderate, and R_s_ > 0.80 strong [40]. In addition, for the behavioral parameters in the individual tests, cows below the 25% quartile were categorized as “low” and cows over the 75% quartile were categorized as “high” [41]. To test whether the cows in these categories differed in view to their object contact duration in the group context, we compared the group test results for the “high” and “low” categories using Wilcoxon’s rank-sum test.

**Stability over time:**The stability of the behavioral test parameters over time was determined for the individual test and for the group test using Spearman’s rank correlation coefficients.

#### Personality traits

The following analyses were performed using the R package psych [42]. We obtained personality traits via PCA with varimax rotation. PCA was performed using the seven individual test parameters obtained for the 39 cows tested in the spring. The suitability of our data set for PCA was confirmed with the measure of sampling adequacy using the Kayser–Meyer–Olkin criterion and Bartlett’s sphericity test [39]. We used the Spearman’s rank correlation matrix as input data (S3 Table) (following [23]), because the behavioral parameters were partly not normally distributed. Given the small sample size, we performed two additional PCAs to assess the component stability. In this analysis, we used the separate spring and autumn individual test results for the 33 cows that we tested twice. Tucker’s congruence coefficient [43] was calculated using the loading matrices from all three PCAs to determine the similarity of the components from the different PCAs. The number of rotated components (RC) for extraction was determined by the Kaiser rule (components with an eigenvalue >1) and using Horn’s parallel test [39]. We used the weights obtained together with the standardized behavioral parameters to calculate the RC scores for each cow. Furthermore, these weights were also used to predict the RC scores for the 33 cows that we retested in the autumn.

**Stability between contexts:** To test the manifestation of the measured personality traits in the group context, we calculated Spearman’s rank correlation coefficients between the RC scores and group test results. In addition, cows, that exhibited a clear behavioral tendency for each personality trait were categorized as low (RC scores < –0.5) or high (RC scores > 0.5) according to a previously published definition [23]. We then compared the group test situation results for the cows in these categories using Wilcoxon’s rank-sum test.

**Stability over time:** The temporal stability of an individual in terms of each personality trait was measured with the Spearman’s rank correlation coefficient based on the corresponding spring and autumn RC scores. In addition, to test the stability of individuals considering both personality traits, we calculated the distance (in standard deviations (SDs)) between the spring and autumn scores within the two-dimensional space. Using these distances, the cows were classified into three classes: distance < 1 SD, 1–2 SD, and > 2 SD (following [23]).

## Results

### Behavioral parameters

Descriptive statistics for the behavioral parameters are provided in S4 Table. The individual test parameter comprising object look was affected by parity in both seasons and by test day nested in parity in the spring (parity, spring: DF = 2, F = 21.21, *P* < 0.0001; autumn: DF = 3, F = 3.61, *P* = 0.041; test day nested in parity, spring: DF = 16, F = 9.69, *P* < 0.0001; autumn: DF = 15, F = 1.24, *P* = 0.348). This effect was due to the high object look value of one first parity cow in both seasons. We did not apply any correction because none of the other individual test parameters were affected and the sample size was small, with only four cows in their first parity (S1 Table). The analysis did not detect any significant effect of parity or group nested in parity for the group test parameter.

#### Stability between contexts

**Fig 1:**
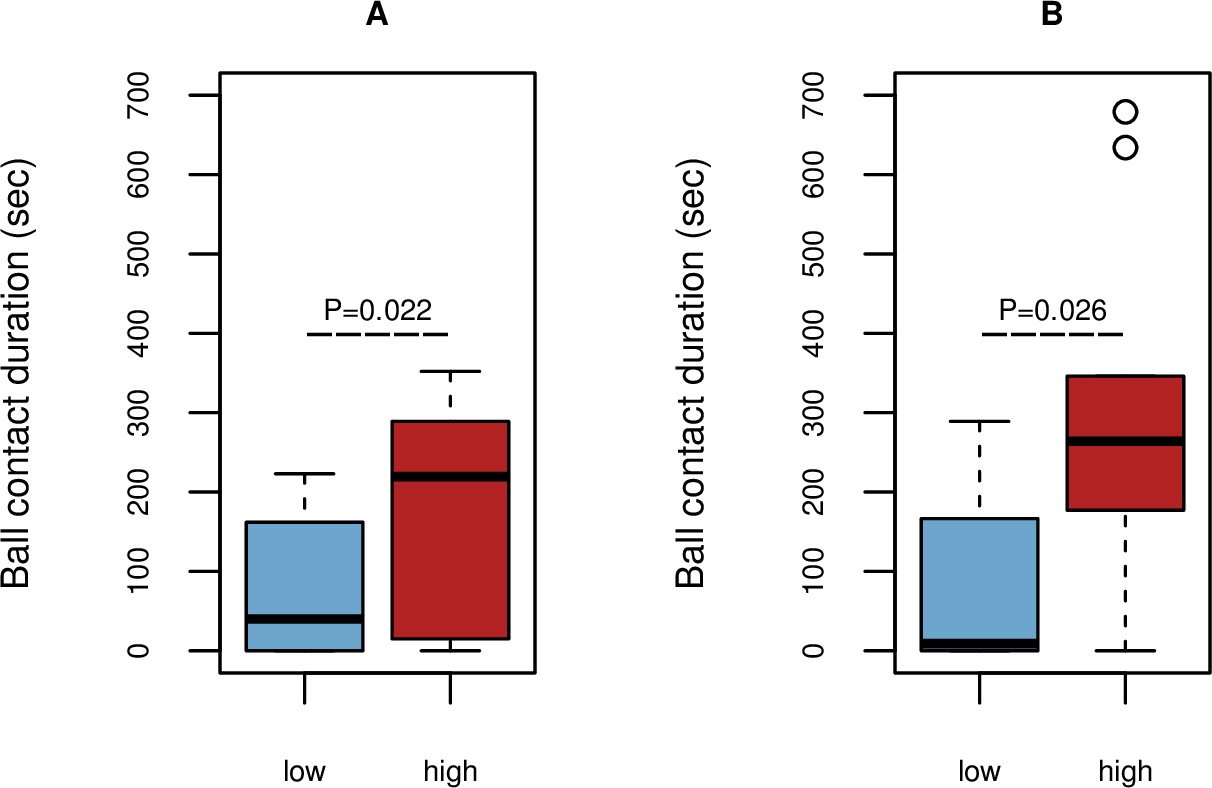
Object contact durations of cows in the group test. Cows were categorized as low (< 25% quartile) and high (> 75% quartile) based on the parameters measured in the individual test: (A) movement duration, (B) object contact duration.

There was a significant positive correlation between object contact in the group test and object contact (R_s_ = 0.361, *P* = 0.026) as well as movement (R_s_ = 0.336, *P* = 0.039) in the individual test. None of the other individual test parameters had significant correlations with object contact in the group test. Hence, we only used the individual test parameters comprising movement and object contact to classify cows as “low” or “high” and we tested for significant differences in the group test results. The corresponding boxplots are presented in Fig 1, which shows that both parameters had significant differences (*P* = 0.022 for movement and *P* = 0.026 for object contact). Cows classified as “high” by movement and “high” by object contact had longer object contacts in the group test than cows in the corresponding “low” categories.

#### Stability over time

In the individual test, the stability between test repetitions was moderate for movement (R_s_ = 0.422, *P* = 0.015), exploration (R_s_ = 0.401, *P* = 0.021), and human contact (R_s_ = 0.569, *P* = 0.001) and low for human look (R_s_ = 0.389, *P* = 0.025). The association between the spring and autumn results was negligible for the other behavioral parameters (range: R_s_ = 0.112–0.246). For the group test, there was no correlation (R_s_ = –0.025, *P* = 0.906) between the spring and autumn results for the 25 cows that we tested twice.

### Personality traits

PCA was applicable because Bartlett’s sphericity test rejected the hypothesis of all zero correlations (*P* < 0.001) and the measure of sampling adequacy was > 0.5 in all cases [39] (0.552 for spring, 39 cows; 0.575 for spring, 33 cows; 0.575 for autumn, 33 cows). In the spring PCA, three components had eigenvalues > 1, but only two in the autumn PCA. In addition, simulations using Horn’s parallel test indicated the extraction of two components (S2 Fig). Based on these test results, two RCs were extracted.

The results of the three PCAs are presented in Table 2. In the first PCA (using all of the cows tested in the spring), the two extracted RCs explained 54.9% of the total variance. In the two other PCAs, using the spring and autumn values for the 33 cows that we tested twice, the RCs explained 53.1% and 55.4% of the total variance, respectively. The similarity of the corresponding RCs obtained from the three PCAs was assessed with Tucker’s congruence coefficient, which indicated good similarity for all pairs (all values >0.94; S5 Table). Furthermore, the Spearman’s correlation coefficients between the RC scores from the two spring PCAs and between the predicted scores and autumn PCA scores were all higher than 0.95. We assigned personality trait names to the RCs based on the biological meanings of the behavioral parameters with “very good” loadings at least (>0.63 or < –0.63) [44]. RC1 was determined by the loadings for movement, exploration, and mirror, and it was termed activity/exploration. RC2 was determined by the loadings for object contact and human contact in spring and human look and human contact in autumn, and thus it was designated as boldness (Table 2).

**Table 2:** Loadings for the behavioral parameters and personality traits assigned to the obtained rotated components (RC).

	Spring (39 cows)		Spring (33 cows)		Autumn (33 cows)	
Parameter	RC1	RC2	RC1	RC2	RC1	RC2
	(32.4%)	(22.5%)	(32.1%)	(21.0%)	(33.7%)	(21.7%)
Movement	*0*.*919*	0.143	*0*.*922*	0.138	*0*.*876*	0.077
Exploration	*0*.*874*	–0.103	*0*.*838*	–0.215	*0*.*874*	0.050
Mirror	*–0*.*755*	–0.180	*–0*.*761*	–0.191	*–0*.*768*	–0.013
Object Look	–0.205	–0.486	–0.235	-0.331	–0.329	–0.345
Object Contact	–0.122	*0*.*669*	–0.124	*0*.*688*	0.299	0.562
Human Look	–0.006	–0.430	0.020	–0.478	0.198	*–0*.*715*
Human Contact	0.187	*0*.*800*	0.210	*0*.*748*	0.044	*0*.*753*
**Personality Trait**	Activity/ Exploration	Boldness	Activity/ Exploration	Boldness	Activity/ Exploration	Boldness

The percentage of variance explained for each RC is shown in parentheses. Parameters with high loadings (>0.63 or < –0.63) are shown in italics.

#### Stability between contexts

In the spring, there was a weak positive correlation between object contact duration in the group test and the RC2 scores for the cows (R_s_ = 0.302, *P* = 0.065). In contrast to the classification using single test parameters, the classification based on RC scores as high and low did not indicate significant differences.

#### Stability over time

**Fig 2:**
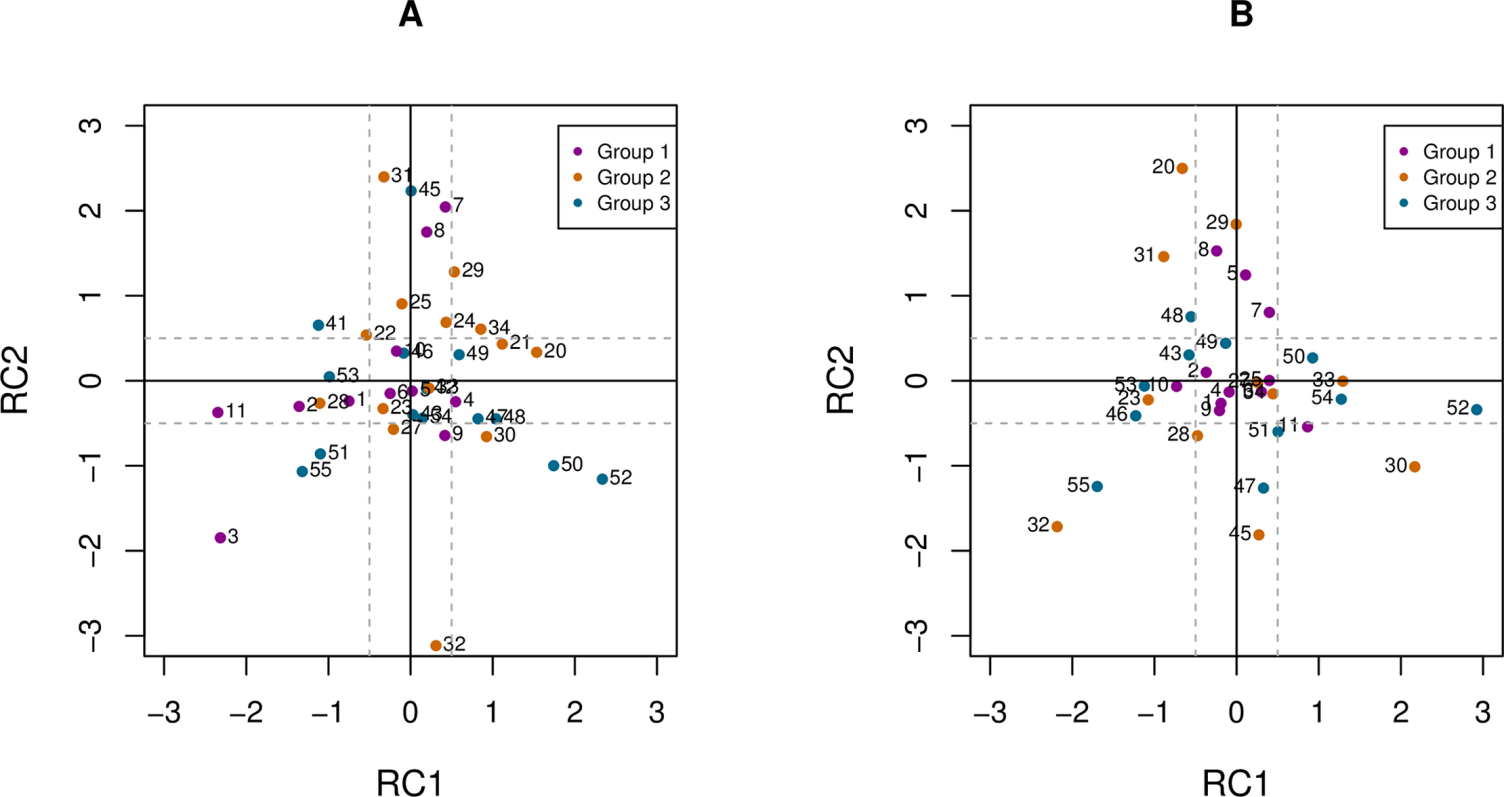
**Rotated component (RC1 and RC2) scores for cows in the spring (A; 39 cows) and predicted scores in the autumn (B; 33 cows).** The analysis was performed in each season based on all cows, colors highlight the group assignment of cows.

The positions of cows within the two-dimensional space based on the PCA conducted with 39 cows in the spring and the predicted RC scores in the autumn are presented in Fig 2.

Considering the stability within the two-dimensional space, in the repeated test 48.5% of the cows scored < 1 SD, 39.4% between 1–2 SD, and 12.1% < 3 SD distance from their spring scores (S3 Fig).

**Fig 3:**
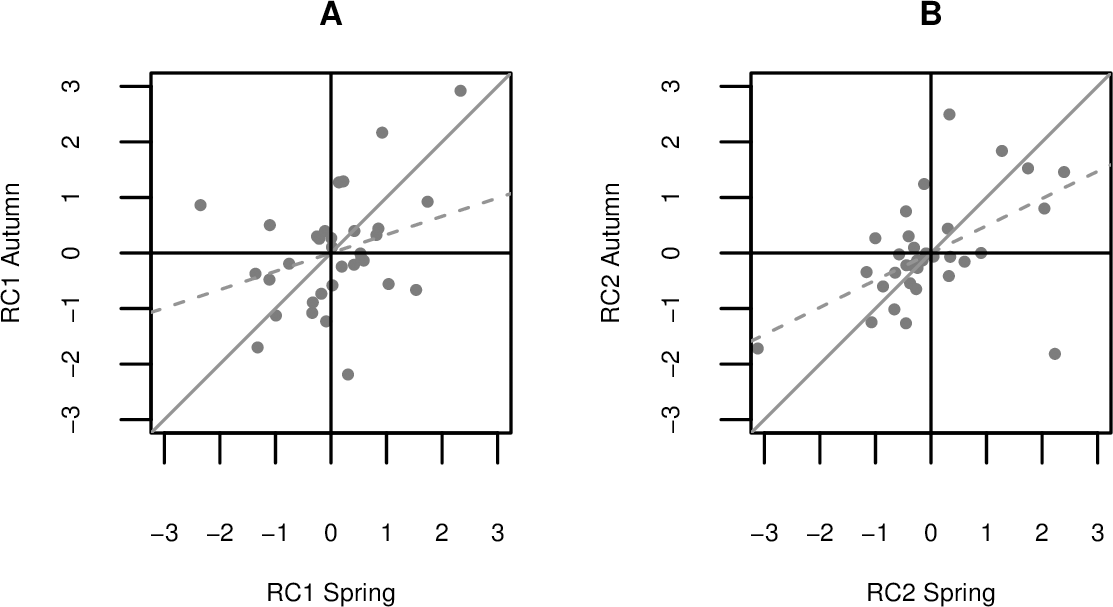
**Stability of the rotated component (RC) scores between spring and autumn for (A) RC1 and (B) RC2.** Solid gray line represents 100% stability between tests. Dashed gray line is the trend line.

The stability of the RC scores between spring and autumn is shown in Fig 3. Not all of the individual test parameters were repeatable, but we found a positive association for both of the derived personality traits, where the correlations between the spring and autumn RC scores for cows were R_s_ = 0.334 (*P* = 0.058) for RC1 (activity/exploration) and R_s_ = 0.491 (*P* = 0.004) for RC2 (boldness).

## Discussion

By definition, personality traits are individual behavioral characteristics that exhibit consistency over time and between contexts [13]. To obtain a better understanding of the contextual and temporal stability of behavior in adult lactating dairy cattle, we measured the consistency of behavioral parameters obtained in repeated individual and group test situations. In addition, individual arena test parameters were used to derive multiple personality traits via PCA. The stability of these personality traits over 6 months and their agreement with the group test results were also investigated.

### Behavioral parameters

Open field, novel object, and novel human tests have been used to assess behavioral variation in several species [18,29]. The sample size limits the number of variables that can be used for certain statistical analyses, such as PCA [39], so it is usual to discard some of the measured parameters from the analysis in animal personality research. To obtain parameters that provided the best possible descriptors of individual behavioral variation in the arena test, we recorded the commonly used parameters and subsequently applied a reduction procedure. Our goal was to use parameters that had the strongest relationships with the reactions of cows to a new situation. Furthermore, we selected parameters with possibly high variance in order to identify the characteristics of the behavioral reactions that differed between individuals (Table 1).

#### Stability between contexts

In addition to the individual test, we performed a group novel object test within the home pen to measure whether this simple to use test could determine the same individual differences that are routinely measured in time-consuming individual tests. Behavioral tests that are performed in every-day environments might have more practical relevance but they can be influenced by factors that are difficult to control, e.g.,the presence of group companions may lead to social facilitation [45,46] or they may hinder the access to the test object. Nevertheless, we found a positive association between object contact duration in the group test and movement duration in the NA test or object contact duration in the NO test (Fig 1). In a recent study, the novel object contact duration of calves was found to be moderately correlated with the feed variety preference in a forage test when the same animals were tested in the home pen as weaned heifers (however, heifers were tested one by one, while the other group members were held in another section of the pen) [47]. These results and those obtained in other studies [28,31] indicate that some aspects of the behavioral variations that can be observed in an individual test situation are also manifested in the group. In the future, repeated tests using a range of different stimuli (as suggested in [48]) might help to capture the consistent behavioral variations exhibited in the every-day lives of cattle.

#### Stability over time

Four out of the seven individual test parameters showed agreement in the two tests conducted 6 months apart. These were both parameters related to the reaction to the new environment (movement duration and exploration duration) as well as parameters in the context with the appearance of an unknown person (human contact duration and human look duration). It is possible that the NA and NH parts of the arena test were the most stressful, and this might explain the stability of the reactions. In contrast to the NA and NH tests, the parameters measured in the NO test had negligible repeatability. Other studies obtained mixed results regarding the repeatability of the NO and NH test parameters (see [48] for a detailed discussion). It has been suggested that shorter intervals between tests and presenting the same object in the second test will generally improve the repeatability of the test results [48]. However, if our goal is to obtain robust measures for describing the behavioral variation that is consistent over a longer time period, then it may be more beneficial to use personality traits derived from different behavioral parameters obtained in several tests.

The group test results were not consistent in the spring and autumn. In this context, it is important to note that the group composition changed between the two tests (S2 Table) due to calving. We could not assess the impact of cows that had their first test in the autumn, but it was possible to compare the group test results for 25 cows that we tested twice. Importantly, habituation could have caused the inconsistency of the results for the repeatedly tested cows because the object contact duration was considerably shorter in the autumn than in the spring (S4 Table). Similar habituation effects were found in a previous study with a repeated visual obstacle test using lactating cows, which was also conducted in a familiar environment [49]. In addition to habituation, the social environment might affect behavioral variations even in non-social behavior due to carry-over effects [50]. In our study, the number of cows in one group was smaller in the second test (S2 Table). Thus, in this group, the cows experienced less competition for other resources (feeder, lying stalls) in the autumn, which may made more energy available for exploring the novel object. Our video observations in this group also suggest that the behavior of the dominant animals may have affected the group test results. Based on the individual values in this group (S2 Table), it is possible to speculate that two dominant animals may have blocked the object in the first test. Therefore in addition to the other reasons mentioned above, the presence or absence of specific cows in the autumn could explain the instability, thereby indicating the impact of the social structure on the expression of individual behavior.

### Personality traits

The arena test comprised a combination of commonly used individual behavioral tests (NA, NO, and NH) and it represented a stressful situation for the cows [27,51]. We expected that the behavioral reactions in the test situations would differ between individuals according to their personalities. We used different parameters from all three parts of the arena test in a PCA to determine the underlying structure of the behavioral variations. These behavioral parameters were selected using a systematic reduction procedure to maintain the suggested five animals to parameter ratio, which is considered to be the minimum for using PCA [39]. PCA identified two main components, which were confirmed by two additional PCAs based on the spring and autumn results from the retested cows (Table 2). These results indicate that the two extracted components were stable although our data sets were small. These findings support the multidimensional nature of cattle personality described previously in calves [22–24] and they also indicate a stable personality trait structure in adult dairy cattle.

RC1 corresponded to the amount of time a cow spent with locomotion and exploration during the NA test. The mirror behavioral parameter, which was interpreted as inactive behavior because it comprised the time when a cow was standing still and looking in the direction of the one-way mirror in the arena, had a strong negative loading on RC1. Based on the high loading behaviors we associated RC1 with the activity/exploration personality trait. An analogous personality trait was also found in other studies of dairy calves [22,23,52–54] and cows [27]. In the framework proposed by Réale et al. [19], activity and exploration are considered to be different personality traits, but various other studies in cattle have shown that exploration and locomotion in a new environment loaded highly on the same component [23,27,55]. Overall, these findings indicate that these behaviors could have the same motivational background. In addition, locomotion by cows within a test arena was also suggested to represent fearfulness [27,28]. However, the reaction to an alarming situation can also be influenced by the coping style [56] of the animal, and fear may result in different (active or inactive) behavioral responses. Further investigations are required to determine whether these traits in dairy cattle are independent or linked, and if they form a behavioral syndrome. In our study, there was only a weak association between the RC1 scores obtained in the spring and autumn. Habituation to the test situation can lead to decreased locomotion and exploration in a novel environment [28,51], and cows do not exhibit dishabituation even after a long period [27]. In our study, the NA test situation remained completely unchanged between the test repetitions, so the low stability of the feature measured in this test phase might be explained by habituation.

RC2 was positively associated with the duration of contact with the object or human. Long contacts with the novel object or human correspond to risk-taking behavior, and thus we associated RC2 with the boldness personality trait, which is described as the propensity to take risks [17,57]. The interpretation of this personality trait on the shyness–boldness continuum was further supported by the negative loadings of the parameters comprising object look duration and human look duration. Our results indicated that some shy cows looked for long periods at the unknown object or human, but they had little contact. The level of attention to the potential source of danger may also be determined by the anxiety of the cow as a distinct trait as well as by its boldness, which could explain the weak negative loadings for these parameters on RC2. We detected moderate stability of boldness after a 6 month period (Fig 3), thereby indicating the practical relevance of this trait in adult dairy cattle. These findings agree with previous studies that also identified a corresponding trait in cattle using open field and novel object tests in cows [28] and calves [22,23,54], although these studies employed shorter time periods between test repetitions.

A previous meta-analysis of studies that reported the repeatability of behavioral traits in non-domesticated animals determined an average repeatability of 0.37 [58]. Our results are in the same range for both of the personality traits identified in the present study. In calves, analogous personality traits showed slightly lower temporal stability, and the positions of the calves within the two-dimensional space (cf. S3 Fig) were also less consistent [23]. Behavior and reactivity might be more flexible during early ontogenesis [59,60], which could explain the higher stability that we found in adult dairy cattle compared with calves [61,62].

Despite the connection between the object contact durations in the individual and group tests, the association was weak between the boldness personality trait and novel object contact in the group test. The group test was conducted in the home environment with other group members present, so it was less stressful than the individual arena test, and thus it might have allowed for greater behavioral plasticity [26]. Furthermore, only one behavioral parameter was assessed in the group test, so it was probably less reliable than a personality trait derived from several measures. Automated data collection based on systems such as high-resolution location tracking could be employed in the future to obtain different behavioral parameters under group housing conditions over a longer time period. These measurements may be useful for studying the connections between social behavior and personality, and they might broaden our knowledge on how behavioral variation is shaped by the environment.

## Conclusions

Overall, we found consistency between the single behavioral parameters measured in adult dairy cattle in two different contexts, i.e., individual and group tests. The repeated measurements after 6 months indicated the stability of most of the individual test parameters but not the group test parameter. Furthermore, based on the repeated measurements of individual behavior in a combined arena test, we identified two personality traits comprising activity/exploration and boldness, underlining the multidimensional nature of personality in cattle. These personality traits showed low to moderate stability after 6 months. The behavioral parameter measured in the group test only had a weak correlation with the corresponding personality trait. Overall, our results indicate that there is a relationship between the social environment and the manifestation of personality traits in every-day situations.

## Supporting information

S1 Fig Novel object in the individual arena test (A) and group test (B).
(JPG)

S2 Fig Results of Horn’s parallel test: (A) spring, 39 cows; (B) spring, 33 cows and (C) autumn, 33 cows.
(PDF)

S3 Fig Stability of the rotated component (RC1 and RC2) scores for 33 cows in the two-dimensional space. Positions of cows are represented by a gray square in the spring and by a black triangle in the autumn.
(PDF)

S1 Table Arena test parameters used in the analysis and the corresponding fixed effects for each cow in the spring and autumn. (XLS)

S2 Table **Group novel object test results and fixed effects in spring (left) and autumn (right) for the three groups.** Retested cows are marked in gray. Mean values for the retested cows are indicated by Mean rep.
(XLS)

S3 Table Spearman’s rank correlation matrices based on the individual test parameters used in three different principal component analyses. ** *P* < 0.05, * *P* <0.1
(XLS)

S4 Table Descriptive statistics for the behavioral parameters used in three different principal component analyses and in the group test. All of the behavioral parameters were measured in seconds.
(XLS)

S5 Table Tucker’s congruence coefficients for the similarity of the rotated components (RC1 and RC2) obtained from three different principal component analyses. (XLS)
