# Supplementary figures and images for: Evaluating the temporal and situational consistency of personality traits in adult dairy cattle
Source: PLoS One. 2018 Oct 1;13(10):e0204619. doi: 10.1371/journal.pone.0204619 (PMC6166944; doi:10.1371/journal.pone.0204619)

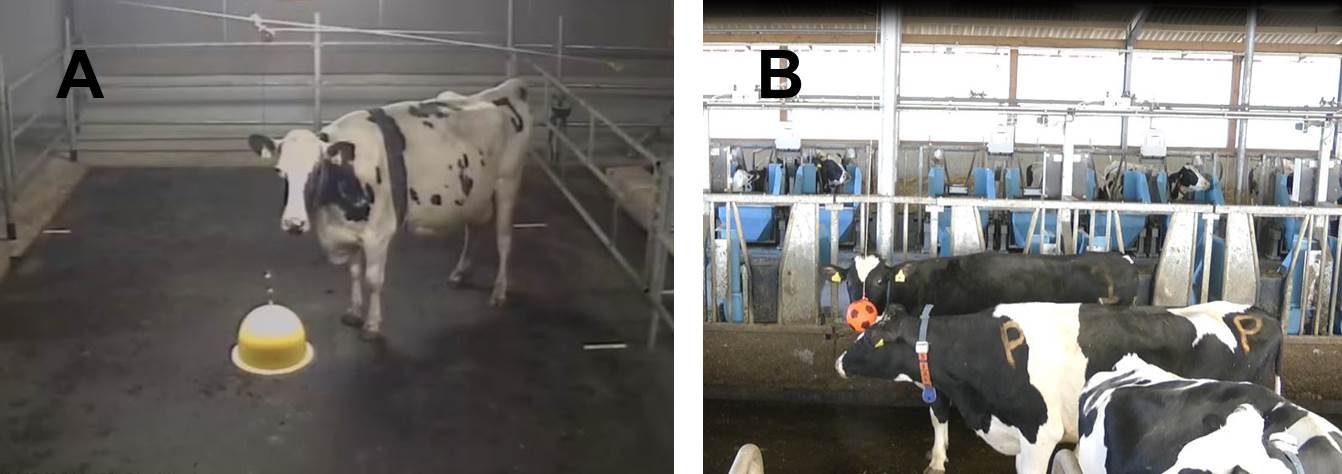

Supplement: S1 Fig — Novel object in the individual arena test (A) and group test (B). (JPG) [file pone.0204619.s001.jpg]

**A**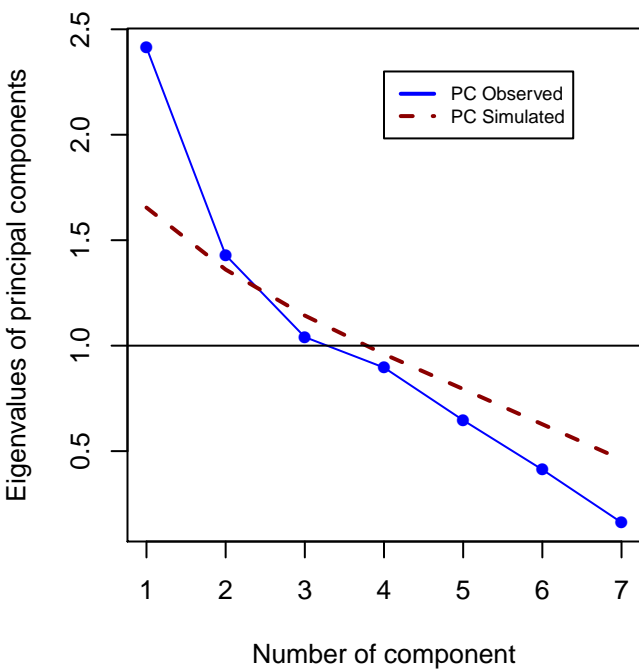**B**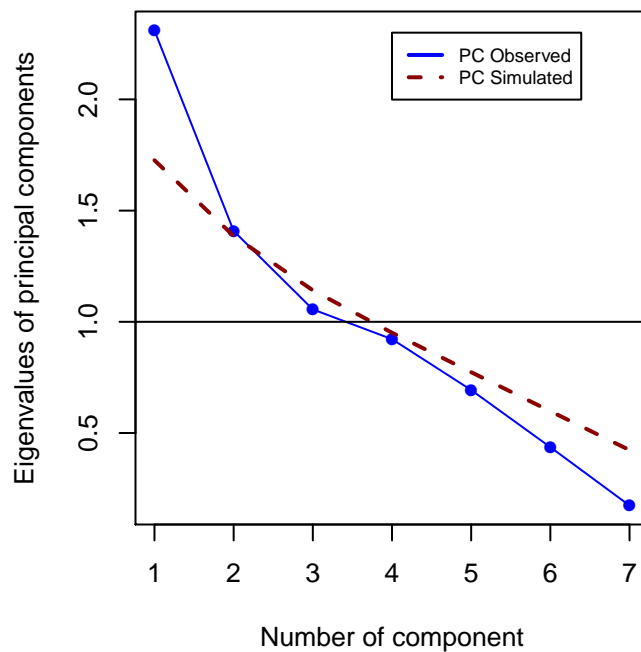**C**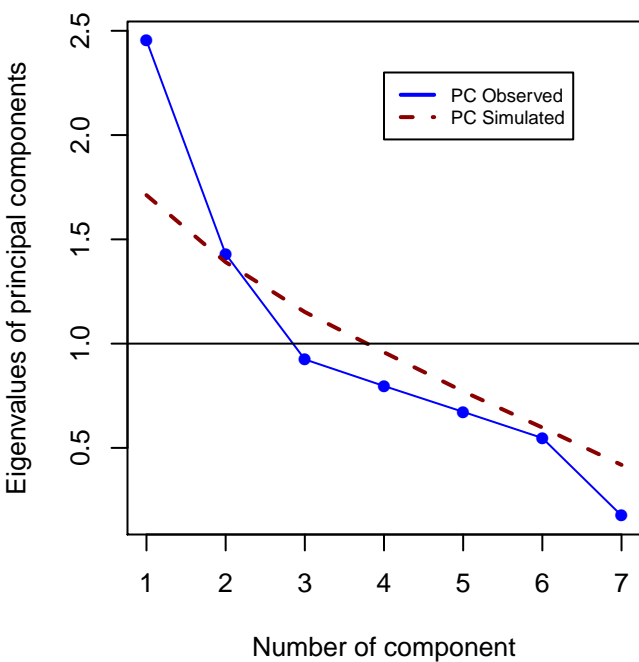

Supplement: S2 Fig — Results of Horn’s parallel test: (A) spring, 39 cows; (B) spring, 33 cows and (C) autumn, 33 cows. (PDF) [file pone.0204619.s002.pdf]

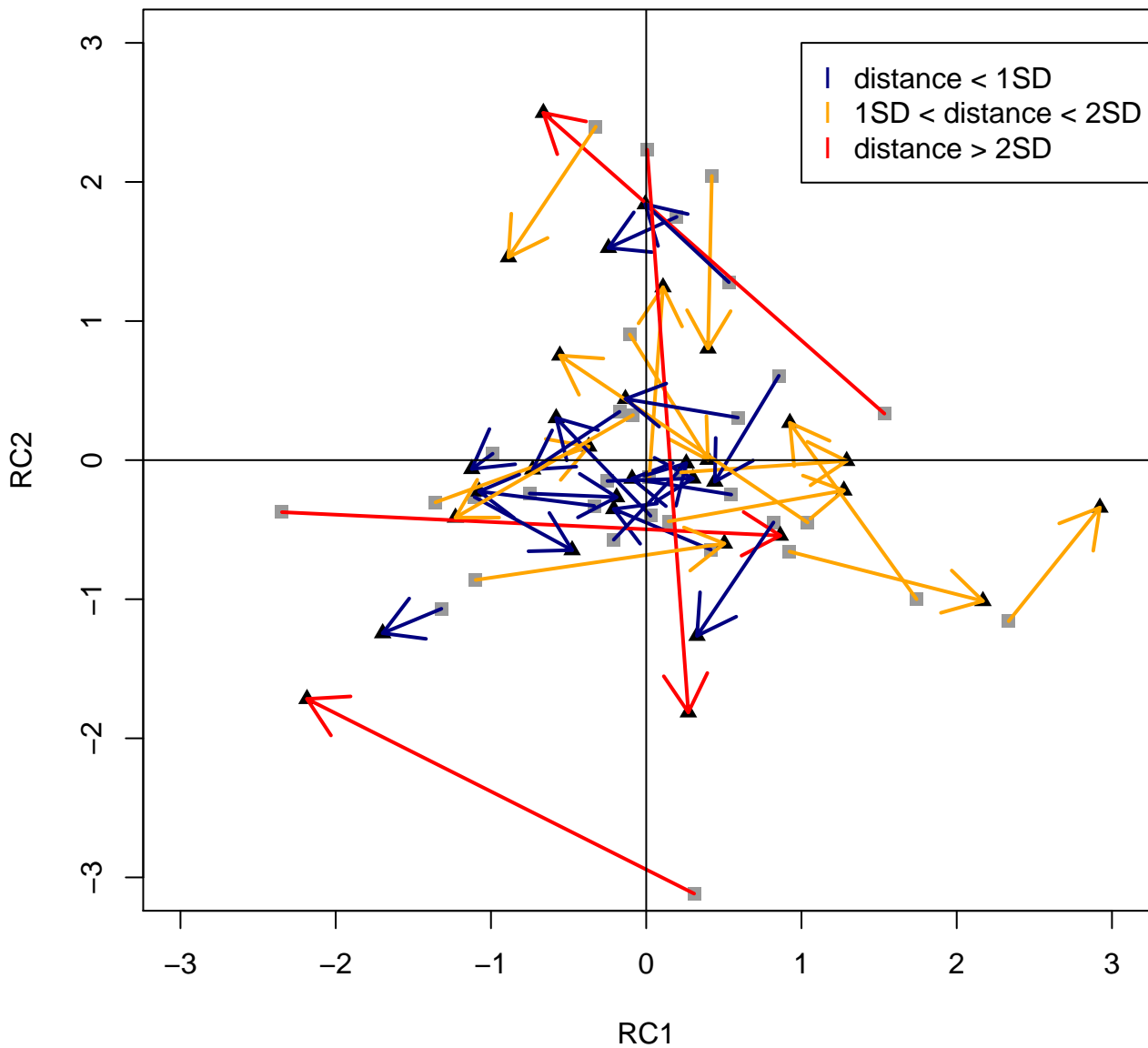

Supplement: S3 Fig — Positions of cows are represented by a gray square in the spring and by a black triangle in the autumn. (PDF) [file pone.0204619.s003.pdf]
